# Non-invasive contrast enhanced MRI tissue characterisation early and late after heart transplantation

**DOI:** 10.1186/1532-429X-14-S1-P180

**Published:** 2012-02-01

**Authors:** Henning Steen, Eva Hofmann, Hugo A Katus

**Affiliations:** 1 Department of Cardiology, University of Heidelberg, Heidelberg, Germany

## Background

Non-invasive MRI tissue characterisation after HTX could potentially replace the hazardous myocardial biopsy for tissue staging and detection of transplant rejection. Recently, late gadolinium contrast enhanced cardiac MRI (LGE-CMR) showed infarct-typical patterns already early after HTX. Additionally, infarct-atypical LGE patterns were also shown in general but neither morphological differentiation nor its time course after HTX have been investigated.

We hypothesized that MRI could detect and differentiate different forms of infarct-atypical LGE patterns and show differences in their morphological appearance during the time course after HTX.

## Methods

123 patients (pts) were divided into group I (62 pts; HTX operation<2ys) and group II (61 pts; HTX operation>2ys). LGE-CMR (Gadolinium:0.2mmol/kg bw) was performed on a 1.5T Whole Body MRI scanner (Philips Medical Systems) and analysed blindly by two experienced observers. For anatomic LGE description, hearts were divided according to the 17-segment model. Areas of infarct-atypical LGE patterns were classified into four types as a) LGE at the RV-insertion (inferoseptal), b) intramural, c) nodular and d) diffuse. Groups were compared using ANOVA. P-values ≤ 0.05 were considered statistically significant.

## Results

**Figure 1:**
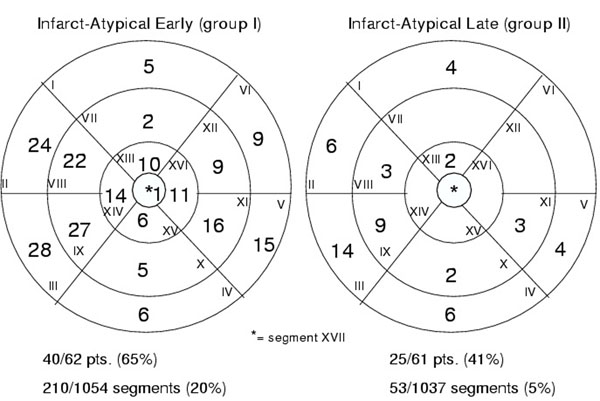
Localisation of early vs. late infarct-atypical LGE-MRI.

**Figure 2:**
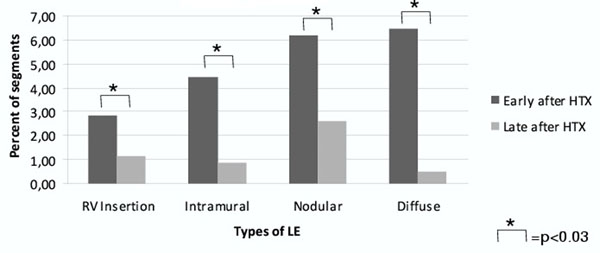
Infarct-atypical LE early and late after HTX.

In group I, 65% of patients and 210 of 1054 segments (20%) showed infarct-atypical LGE patterns. In contrast, significantly less patients (41%) of group II and only 54 of 1037 (both p<0.01) segments were affected (figure 1) apparently omitting the anterior and antero-lateral basal and mid-ventricular segments. All four LGE-CMR types showed significantly higher prevalence (p=0.03) in group I vs. group II (figure 2).

## Conclusions

LGE-CMR is a novel and sensitive imaging technique for myocardial tissue characterisation after HTX. Unfortunately, the exact patho-mechanism for the occurrence of infarct-atypical CE-MRI patterns is illusive but could potentially include the amount of organ rejections, myocarditis or other immune processes. Further studies need to clarify the correlation between myocardial biopsies and the various patterns on MRI images. Nevertheless, LGE-CMR in HTX could be a valuable tool for non-invasive tissue characterisation in pts after HTX and could have the potential to replace invasive biopsy procedures in the future.

## Funding

None.

